# “The sweet and the bitter”: mothers’ experiences of breastfeeding in the early postpartum period: a qualitative exploratory study in China

**DOI:** 10.1186/s13006-020-00256-1

**Published:** 2020-02-24

**Authors:** Xiao Xiao, Alice Yuen Loke, She-ning Zhu, Lin Gong, Hong-mei Shi, Fei-wan Ngai

**Affiliations:** 1 Department of Obstetrics, Affiliated Shenzhen Maternity & Child Healthcare Hospital, Southern Medical University, Shenzhen, China; 2 School of Nursing, The Hong Kong Polytechnic University, Hung Hom, Kowloon, Hong Kong, China; 3 Department of Nursing Administration, Affiliated Shenzhen Maternity & Child Healthcare Hospital, Southern Medical University, Shenzhen, China; 4 Women’s Health Centre, Affiliated Shenzhen Maternity & Child Healthcare Hospital, Southern Medical University, Shenzhen, China

**Keywords:** Breastfeeding, Postpartum, Maternal mental health, Breastfeeding support, Breastfeeding promotion, Breastfeeding initiation

## Abstract

**Background:**

In China, the prevalence of exclusive breastfeeding at 6 months was only 20.8%. In promoting breastfeeding for newborns, a number of strategies have been initiated by Chinese government. These actions facilitated a high breastfeeding initiation of 77 to 99.9% in different regions. However, the exclusive breastfeeding rates remained low at 6 months resulting from a high rate of perceived insufficient breast milk and complementary feeding during the early days after childbirth. The aim of this study was to understand the experiences of women in Shenzhen with regard to breastfeeding in the first 6 weeks after giving birth, to identify the facilitators and barriers impacting their breastfeeding decisions and to identify their perceived support needs that might facilitate breastfeeding in the future.

**Methods:**

This was a qualitative exploratory study. Data were collected in November 2018 through semi-structured, face-to-face, in-depth interviews. A purposive sample of early postpartum women was recruited from a postpartum clinic of a tertiary maternal hospital in Shenzhen, China. The dataset was analysed using inductive content analysis.

**Results:**

A total of 22women were interviewed within the first 6 weeks after delivery. Three themes related to breastfeeding were identified from the transcribed interviews: “breastfeeding facilitators,” “breastfeeding barriers,” and “recommendations for breastfeeding promotion.”

**Conclusions:**

Women experienced both joy and suffering in their journey of breastfeeding. Insufficient knowledge of breastfeeding, discomfort, intergenerational disagreements regarding nutritional supplements, and a lack of professional support contributed to difficulties and the threat of discontinuation. A supportive environment for breastfeeding is crucial for women’s decision on exclusive breastfeeding and the psychological wellbeing of breastfeeding women. Interventions that target to promote exclusive breastfeeding should include both new mothers and significant family members. Future studies could test the effectiveness of breastfeeding training for home visit nurses to promote exclusive breastfeeding in the early postpartum.

## Background

In China, the rates and duration of exclusive breastfeeding are still relatively low in many areas. A survey conducted in the central and western regions of China reported a rate of exclusive breastfeeding of 58.3% for newborns (aged 0 to 27 days), which declined to 29.1% at 3–4 months and 13.6% at 5–6 months [1]. The latest national survey in China reported the prevalence of exclusive breastfeeding at 6 months to be 20.8%, and the breastfeeding rate dropped to 11.5% at 1 year and 6.9% at 2 years [2]. These breastfeeding rates fall short of the targets recommended by the World Health Organization (WHO) [3, 4].

China is among the largest infant formula consumption countries [5]. In accordance with the breastfeeding promotion initiative, a number of strategies have been implemented by the Chinese government, such as the Baby Friendly Hospital Initiative, breastfeeding education programmes, women’s and children’s health protection legislation, and social support programmes [6]. Due to the efforts of these actions, the breastfeeding initiation rate ranges from 71.3 to 99.9% in the first month postpartum in various cities in China [7]. However, the exclusive breastfeeding rates at 6 months remain low due to a high rate of perceived insufficient breast milk and early complementary feeding [1, 6]. It has been found that early cessation of exclusive breastfeeding is related to the mother’s early breastfeeding experiences [8]. Infrequent breastfeeding during the early postpartum period and delayed breastfeeding initiation are associated with a lower rate of exclusive breastfeeding and early cessation of breastfeeding [8]. In addition, early breastfeeding experiences play a significant role in establishing mothers’ feeding behaviours [8]. For example, in some areas in China, it is believed that infants should not be breastfed for some time after birth [9]. A recent survey reported that the first feed of more than 60% babies was infant formula [10]. About 70% of infants were first breastfed 24 h after birth [10, 11]. Only approximately one out of ten women initiates breastfeeding within the first hour of delivery, as recommended by the WHO [11, 12]. Thus, to improve the rate of exclusive breastfeeding and breastfeeding duration, it is important to understand Chinese women’s early breastfeeding experiences.

### Experience of mothers in initiating and continuing breastfeeding

According to the conceptual model proposed by Rollins et al., women’s decisions and experiences related to breastfeeding are influenced by the sociocultural context, settings (including health systems and services, family and community, workplace and employment) and individual perceptions of breastfeeding [5].

A systematic review in South Asia noted that traditional feeding practices, the availability and accessibility of breastfeeding information, insufficient breast milk, and inadequate health services were the common barriers to exclusive breastfeeding [13]. In Chinese culture, mothers feel embarrassed and unwilling to breastfeed publicly because female breasts are regarded as sexual objects, and it is shameful for Chinese mothers to expose their breasts in public [14]. Therefore, mothers feed their babies with expressed milk in bottles or supplement with infant formula when they are outside the home [14].

The experiences, constraints, and difficulties that women anticipate or encounter while breastfeeding may impede their initiation and continuation of exclusive breastfeeding or lead to breastfeeding cessation [5]. The difficulty of getting the baby to latch, sore or painful nipples, or insufficient milk production are common reasons for discontinuing exclusive breastfeeding [15]. Breastfeeding problems are more likely to occur in the early weeks after birth [16, 17]. A Danish study found that more than 40% of mothers experience early breastfeeding difficulties [16]. This rate was much higher (92%) in a study in California [18]. In addition to frequent breastfeeding problems, inadequate support is provided to breastfeeding women in the early postpartum period [16]. Limited studies have reported the incidence of breastfeeding problems among Chinese mothers in the early postpartum period. Thus, further research is required to explore this situation in China.

The prevailing public health messages and the emphasis of health professionals on the advantages of breastfeeding have pressured women to breastfeed their infants. Many women may also feel obliged to breastfeed because of family or social expectations [19, 20]. A study in Turkey described women being ordered to breastfeed their babies by older family members and being influenced by religious beliefs [21]. A study in the United Kingdom (UK) reported that women struggled to meet the expectation to breastfeed to avoid being judged as not a good mother [19]. However, those who did not enjoy breastfeeding discontinued the practice after a relatively short time and felt guilty for doing so. Once women stopped breastfeeding, they were less likely to initiate breastfeeding in the future [17]. A recent study noted that women’s willingness to breastfeed was also associated with their postpartum depressive symptoms [22]. Women who had planned to breastfeed but were unable were more likely to be depressed [22].

Mothers’ feeding practices may also be influenced by their immediate social networks [13]. In China, grandmothers’ opinions or involvement in decision making has been correlated with early complementary food supplementation [13]. Mothers or mothers-in-law are reported to persuade new mothers to supplement their breastfeeding with infant formula due to concerns regarding insufficient breast milk production, and these women may discourage the mothers from continuing to breastfeed exclusively [23]. Postpartum women’s emotions are heavily influenced by family conflicts regarding breastfeeding [24]. Legislation and an environment that supports exclusive breastfeeding have been weak in China [25]. Studies have also reported that short maternity leaves, husbands’ opposition to breastfeeding, women’s low self-efficacy with regard to breastfeeding, and the lack of public places to breastfeed have led to a low prevalence of exclusive breastfeeding among women in Hong Kong [26, 27].

In response to the WHO’s call to reach a global rate by 2025 of 50% of mothers breastfeeding exclusively at 6 months postpartum, strategies must be developed to encourage and facilitate the initiation and continuation of breastfeeding. China is one of the largest infant formula consuming countries [2], and understanding women’s breastfeeding experiences in the early postpartum period can be helpful by enabling health professionals to provide more meaningful support to postpartum women. There is currently a lack of understanding of women’s early breastfeeding experiences during their breastfeeding journey.

#### Research aims and objectives

The aims of this study are three-fold:

1. To understand women’s breastfeeding experiences in the early postpartum period (6 weeks postpartum);
2. To identify the facilitators of and barriers to the decision to breastfeed exclusively; and
3. To identify women’s perceived needs for support that might facilitate breastfeeding and to make recommendations to promote the continuation of breastfeeding in Shenzhen, China.

## Methods

### Study context

This study was conducted in a tertiary maternal hospital in Shenzhen. The hospital received accreditation in 2016 as a national baby-friendly hospital. The hospital targets a breastfeeding initiation rate of 100% and encourages exclusive breastfeeding for 6 months and the continuation of breastfeeding for 2 years. Strategies adopted in this hospital to promote breastfeeding include antenatal education for expectant mothers, one-to-one bedside advocacy and support for women seeking to breastfeed after delivering in the hospital, and postpartum home visits after discharge.

### Study design

An exploratory case study design was used in this study [28]. This study aimed to understand the phenomenon of women’s breastfeeding experiences, identify how women made decisions regarding breastfeeding, and examine why women chose exclusive breastfeeding or not exclusive breastfeeding during the early postpartum period in Shenzhen, China. Previous studies have found that the exclusive breastfeeding rate is much lower in large cities than in suburbs or rural areas in China [9]. Shenzhen is one of the top four cities in China, but the rate of exclusive breastfeeding and the rate of any breastfeeding in the first month remain among the lowest levels [7].

Shenzhen is an immigrant city, with 67.7% of residents originally from other parts of China [29]. It is a common practice for women to stay at home and rest, be cared for by their mother or mother-in-law and to co-reside together during the postpartum period. Older generations who favour traditional feeding practices or those from different cultures affect women’s breastfeeding decisions and experiences [9]. The first 4 to 6 weeks after birth are regarded as the most significant time for breastfeeding cessation or the establishment of exclusive breastfeeding [30]. The understanding of postpartum women’s breastfeeding experiences was interpreted based on the researcher’s experience of living and working with postpartum women in Shenzhen.

### Participants

To obtain a comprehensive understanding of the research question, a purposive sampling strategy was employed to recruit suitable participants. Because mothers in the early postpartum period experience the most problems with breastfeeding [31], this study focused on the breastfeeding experiences of women in the first 6 weeks after delivery. It was expected that first-time mothers would experience more breastfeeding problems and that non-first-time mothers would be influenced by their past experiences of breastfeeding [32]. First-time mothers and experienced mothers were recruited into this study. The criteria for inclusion in this study were as follows: (1) women who had given birth to a singleton baby in the last 6 weeks; (2) a full-term delivery of between 37 and 42 weeks; (3) baby’s birth weight ≥ 2.5 kg and 5-min Apgar ≥8; (4) baby not admitted to the Neonatal Intensive Care Unit and with no abnormalities; and (5) baby on any feeding regimen (a combination of breast milk and other types of milk or food or drink) or exclusively breastfed in the last 6 weeks. Women were excluded if they had any reported mental health problems or any postpartum complications. The sample size of the participants was determined by data saturation.

### Data collection

Data were collected in November 2018 through semi-structured face-to-face interviews. Women who came to the women’s health centre for a postpartum check-up at 30 or 42 days and who met the inclusion criteria were invited to take part in the study. The purpose of the study was explained to them, and the women who consented were interviewed in a quiet room in the health centre.

The first author conducted the in-depth interviews. All of the interviews were audio-taped, and field notes were recorded. The participants’ demographic information was collected via a demographic questionnaire. The questions for the semi-structured interviews were compiled by the researcher based on her experience working with postpartum women and were discussed with two academics in the fields of obstetrics and family nursing. The opening question for the interview was, “Can you tell me your experience of feeding your baby?” Probing questions were then used to obtain in-depth information or further clarifications, such as, “Have you ever encountered any difficulties in breastfeeding?” When women reported enjoyable breastfeeding experiences, they were asked to provide details. Each interview lasted 25 to 40 min. The process of data collection was supervised and checked by two experienced researchers. Data collection continued until no new information emerged from the interviews. Two additional participants were interviewed to confirm that data saturation had been reached. By the 20th participant, no new information was identified.

### Data analysis

Inductive qualitative content analysis was used to analyse the data [33]. Field notes were also taken during the interviews [34]. Data analysis was conducted concurrently with data collection. Each recording was transcribed verbatim within a week of the interview, and the accuracy of the transcripts was checked by two other researchers on the team. Each transcript was read several times by two researchers, and the categories were identified independently. A category was identified and extracted if it was related to the criterion and the research question [33]. Formulated meanings were clustered into new categories. The categories and transcripts were compared for similarities and differences.

### Ethical approval

Ethical approval for this study was obtained from the Ethics Boards of the Hong Kong Polytechnic University (Reference no. HSEARS20181015003) and the Shenzhen Maternity and Child Healthcare Hospital (Reference no. 2018278). Written informed consent was obtained from the participants in the process of recruitment before the interviews commenced.

### Rigor

All interviews were audio-taped and transcribed verbatim to ensure the credibility of this study. Field notes were taken and considered during the data analysis. During the data analysis, the researchers employed a bracket strategy and put their own views aside to avoid personal bias. Regarding conformability, the authors held discussions on a coding scheme and identifying themes until consensus was achieved. If they could not reach consensus, a third researcher was invited to engage in further discussion. An audit trail was conducted. Two participants were asked to read the findings to confirm that the findings were in accordance with their narration.

## Results

A total of 22 women were interviewed. Among them, 14 (63.6%) were first-time mothers and 8 (36.4%) were second-time mothers. Nine (40.9%) mothers breastfed exclusively, while 13 (59.1%) breastfed partially. The mothers were between the ages of 22 and 43. The demographic details of the women included in this study are presented in Table 1.

**Table 1:** Description of the early postpartum mothers (*n* = 22)

Characteristics	Number
Parity	
Primipara	14
Multipara	8
Education level	
High school	1
Diploma	6
Bachelor’s degree	11
Master’s degree	4
Mode of delivery	
Vaginal delivery	12
Assisted vaginal delivery	1
Planned Caesarean section	6
Emergency Caesarean section	3
Mode of feeding	
Exclusive breastfeeding	13
Mixed feeding	9
Education level of main caregivers	
Middle school or below	8
Secondary school	3
High school	5
Diploma	4
Bachelor’s degree	2

In accordance with the research aims of this study, after analysing the content of the transcriptions, three themes were identified: breastfeeding facilitators, breastfeeding barriers, and recommendations for breastfeeding promotion (Table 2).

**Table 2:** Summary of themes and categories of the findings

Themes	Categories
Breastfeeding facilitators	• Determined to give the baby the best by breastfeeding • Positive breastfeeding experiences • ‘Strategies’ employed to continue breastfeeding
Breastfeeding barriers	• Lack of breastfeeding skills • Constrained life • Tiring and upsetting • Feel guilty for not having sufficient breast milk • Negative breastfeeding experiences • Pressure from family related to breastfeeding • Lack of facilities for breastfeeding in public
Recommendations for breastfeeding promotion	• More breastfeeding information from professionals • Professional support at home

### Breastfeeding facilitators

Breastfeeding was a priority for all of the women interviewed. Women’s positive perceptions of breastfeeding constituted one of their greatest motivations for initiating breastfeeding. They believed that breastfeeding is an inherent part of motherhood and that it was an enjoyable experience to interact with the baby during breastfeeding. They therefore chose to breastfeed their infant and tried several methods to stimulate the production of breast milk.

#### Determined to give their baby the best by breastfeeding

The participants clearly realized the benefits of breastfeeding. The most commonly cited reason for breastfeeding was that it would give their infant stronger immunity. Therefore, women decided that breastfeeding their baby was a priority and that using infant formula was a suboptimal option only to be resorted to when efforts to breastfeed were unsuccessful:

*I prefer breastfeeding, so I do not want to supplement formula for her. We all know that babies who are breastfed will have a stronger body.* (P17, first-time mother)

*I decided to breastfeed my babies. I insisted on breastfeeding no matter how difficult it might be. I believe that breastfeeding is optimal. Only if I had no choice would I supplement feeding with formula.* (P14, second-time mother)

#### Positive breastfeeding experiences

Women who were successful at breastfeeding were satisfied with the experience. The intimate interaction with the infant during breastfeeding strengthened the bond between mother and child and gave the mother a feeling of satisfaction. Women felt that breastfeeding gave them a wonderful feeling of motherhood and that holding the baby in their arms to feed reminded them of their role as a mother:

*It was a blessing to be able to breastfeed. I have intimate moments with my baby. I felt satisfied and happy to see his face while feeding, and he seems contented.* (P16, first-time mother)

*I feel the attachment between me and my baby during breastfeeding (laughter). I feel a stronger attachment to my baby each time I breastfeed her; it gives me the feeling of being a mother*. (P20, first-time mother)

One woman was amazed at the wonder of breastfeeding and that a newborn baby would have the instinct to turn to her breast and start sucking at birth.

*It was amazing the first time I fed her. She actually turned her head to search and latched onto my breast and started sucking right after birth. She was so clever that she would do the same with the other breast too. Nature is just incredible.* (P10, first-time mother)

Other women described their sense of accomplishment when breastfeeding. They regarded themselves as competent mothers because they could breastfeed their babies.

*I have a sense of accomplishment when breastfeeding her. The act of breastfeeding also comforts me in a psychological sense. I feel competent in being able to give what is best for my baby.* (P13, second-time mother)

*My nipples feel a little sore, but when I see her fall asleep after a satisfying meal from breastfeeding, I feel great to be a mother.* (P17, first-time mother)

#### ‘Strategies’ employed to continue breastfeeding

Women regarded breastfeeding as part of the mission of becoming a mother and put a great deal of effort into promoting successful breastfeeding:

*I did not have breast milk in the first few days, so I focused on promoting lactation, and that was my only task at that time. (P16, first-time mother)*

To stimulate lactation to become a “productive mother,” women shared their experiences of trying many different strategies to promote the production of breast milk. Some mothers utilized folk practices, while others relied on nutritional foods / soups to restore their strength and energy to sustain breast milk production.

*I hope to breastfeed my baby, so I have tried to breastfeed. I was hoping that the baby’s sucking would promote breast milk production. I even tried folk practices that I learned from the Internet, which were said to be helpful for stimulating or sustaining lactation. However, I’m disappointed that those practices do not really work. I’m now producing less and less breast milk and will have to switch to formula feeding soon.* (P6, second-time mother)

*My breast milk is not sufficient. I am eating several meals a day and drink a lot to sustain my breast milk production. Otherwise, I don’t think I would have sufficient breast milk.* (P16, first-time mother)

When they did not receive much support from health professionals, women sought the help of a *kainaishi* (a massage therapist who specializes in increasing the amount of breast milk that a mother produces). A *kainaishi* is not a formally trained professional but is traditionally employed to offer breast massages or acupressure to promote the production of breast milk [35].

*We hired a* kainaishi*. She gave me a breast message, which helped me to produce more breast milk. She also provided me with psychological support. I feel relaxed and happy, and I am producing more breast milk.* (P10, first-time mother)

*I called on a* kainaishi *a few times, which helped me to produce more breast milk. I used to produce approximately 30 ml of milk, but this has increased to approximately 70 ml. I need to supplement the feeding with formula.* (P22, first-time mother)

Most mothers were determined to breastfeed their baby and put considerable energy into ensuring that they could produce enough milk for their baby. Women’s experiences, both the positive perception of breastfeeding and the motivation for breastfeeding, facilitated the continuation of breastfeeding.

### Breastfeeding barriers

Women encountered difficulties in the breastfeeding journey that impeded their engagement in the practice. These barriers occurred on the individual, family and organizational levels. At the individual level, they reported not having the skills to breastfeed, which also caused them pain and discomfort. Breastfeeding women felt tired and trapped at home. At the same time, they had to tolerate disturbed sleep and sacrifice their body when continuing to breastfeed. At the family level, family members’ persuasion to supplement with infant formula reduced women’s determination to breastfeed exclusively. In addition, criticism from others regarding insufficient breast milk contributed to the mothers’ sense of guilt. Family conflicts regarding nutritional supplements for breastfeeding also negatively influenced women’s breastfeeding experiences. At the organizational level, the lack of suitable facilities in public places made it inconvenient to breastfeed. These barriers negatively influenced women’s exclusive breastfeeding experiences and decisions.

#### Lack of breastfeeding skills

Many new mothers encountered breastfeeding difficulties. They could turn to nurses for help when they were in the hospital. However, once they were discharged home, they were anxious about being on their own when dealing with breastfeeding.

*In the first few days after discharge, I was totally lost, nervous, and very clumsy when I breastfed.* (P19, first-time mother)

Some of the women were not confident about breastfeeding. Those with short and inverted nipples felt that their baby had problems latching on to a nipple to breastfeed.

*My right nipple is short, so my baby had problems latching on to it. To avoid frustration, I only breastfeed on my left. I do worry that my breasts will be different sizes after breastfeeding.* (P4, first-time mother)

*I don’t know how to feed my baby while sitting. I have to lie down for feeding. I wish someone would teach me how to breastfeed in a sitting position when I hold her in my arms.* (P5, first-time mother)

*I have inverted nipples, so I have pain each time she sucks. I have to use a pump to suck the nipples out before feeding my baby.* (P19, first-time mother)

Discomfort and pain were frequent complaints of breastfeeding mothers. They had to tolerate pain during breastfeeding. They also suffered from sore or cracked nipples or mastitis.

*I felt extreme pain because my right nipple was chapped. I had tried to reduce the frequency of suckling and make sure that my breasts were empty each time.* (P11, second-time mother)

*I had lots of pain when breastfeeding and came to realize that I had mastitis. I still endured the pain and continued breastfeeding. (*P5, first-time mother)

#### Constrained life

Another common theme noted by women was their sense of feeling trapped. They were occupied by the infant if they chose exclusive breastfeeding. Because they thought that it was unacceptable to breastfeed a baby in public places, women felt embarrassed to breastfeed their infant publicly. Enmeshed in the task of breastfeeding the baby, they had to stay at home and wait for the infant to be hungry. Therefore, they were forced to give up their personal social activities and felt constrained.

*I was flexible and could do anything I liked before she was born, but I could not be as free as after the baby was born. I had to bear in mind, ‘I am breastfeeding; forget about hanging out.’ I take the baby into consideration for everything, so I cannot arrange my time as I wish.* (P17, first-time mother)

*I feel like having meals was like fighting in a war . . . I have no fixed time for eating because my needs give way to the baby’s needs. This is completely different from what life was like before his birth. I spend all my time on him and have none for myself.* (P3, first-time mother)

*I would rather not go out because of the inconvenience. It is not acceptable to breastfeed in public.* (P12, first-time mother)

Some women complained that the baby used their breast as a pacifier. They hated the feeling of being trapped.

*She keeps my nipple in her mouth even when she is not hungry and falls asleep. I cannot be free.* (P1, first-time mother)

*I have to breastfeed her all the time. It feels like she sucks forever. She only calms down when she has my nipple in her mouth, or she will start crying.* (P21, first-time mother)

#### Tiring and upsetting

Most women agreed that choosing breastfeeding meant that their sleep was disturbed. They had little time to rest. They were awakened frequently and exhausted from having to feed the baby or to express milk from their breasts to prevent engorgement.

*I have to wake up in the middle of the night. I am either breastfeeding when she is awake or expressing milk when she is sleeping. My breasts are full in two to three hours.* (P7, second-time mother)

Women were torn between having quality breast milk and concerns about their weight. While they wanted to eat enough nutritious food to ensure that they produced quality breast milk, they were also watching their weight.

*I have gained weight with my pregnancy and want to lose weight now, but my nanny told me that I won’t have quality breast milk if I do. I had planned to breastfeed for one year, so I have to wait a year to lose weight and be fat now.* (P20, first-time mother)

*I was on a diet right after I delivered and succeeded in losing 6 kilos. However, this seemed to affect my production of breast milk, which is watery. I have to stop thinking about losing weight because I would like to continue to breastfeed.* (P21, first-time mother)

#### Feeling guilty for not producing sufficient breast milk

Insufficient breast milk was the most common reason reported by women for infant formula supplementation. Steeped in the belief that “a breastfeeding mother is a good mother,” women blamed themselves for not producing sufficient breast milk because they felt that they had failed to give the best to their baby and were incompetent as a mother. They became anxious and started comparing their baby with others.

*I did not produce enough breast milk, and my daughter started to cry without milk. I cannot satisfy the needs of my baby, and I am considering feeding her formula. The paediatrician told me that she is not gaining enough weight. Other babies born around the same day weighed 4 kg, 4.5 kg, or even more, but mine only weighed 3.5 kg.* (P22, first-time mother)

#### Negative breastfeeding experiences

Two new mothers shared their unhappy and extreme experiences of breastfeeding. One mother experienced excessive pain and cracked nipples in the early postnatal period. She described her depression related to her negative breastfeeding experiences.

*Each time I breastfed the baby, it was painful with my cracked and bleeding nipples. I was fearful when the baby was sucking. This caused me to feel depressed. I am feeling a bit better now as my nipples are getting better.* (P17, first-time mother)

Another woman also struggled with breastfeeding. She suffered twice from mastitis. She reflected on her experience and expressed the belief that there was a strong relationship between her mastitis and her bad moods.

*I had mastitis twice and was admitted to the hospital because of a high fever. Each time I breastfed, it was so painful that I cried at night. I screamed and was out of control, and then I was depressed. I had to call an ambulance for help.* (P3, first-time mother)

New mothers are not skillful and encounter difficulties in breastfeeding. They often feel that they are trapped by breastfeeding and easily tire of it. Some of them may also feel guilty if they cannot produce enough milk for their babies. These negative experiences adversely affect new mothers’ psychological wellbeing. Furthermore, excessive pressure from their family and insufficient support from society may hamper their decision to breastfeed exclusively.

#### Pressure from family related to breastfeeding

Women were disturbed that although they were the ones who were breastfeeding their baby, their mothers-in-law focused on cooking them food to boost their production of breast milk. They were pressured to eat food that they did not necessarily believe they should be eating.

*My mother-in-law said that I did not produce sufficient breast milk. She kept cooking her so-called ‘lactation promotion soup’ for me. She cooked ‘loofah’ soup, which is a kind of rough vegetable that we use in dried form to clean dishes. She also cooked ‘pig tongue soup,’ which she believed would promote quality breast milk. I would become malnourished if I followed that diet.* (P10, first-time mother)

In contrast, some women were over-nourished and were concerned about eating different foods. However, all of these concerns about food were related to breastfeeding. There was a gap in knowledge between the two generations.

*My mother-in-law followed the rituals and made me eat at least 6-9 eggs a day. She also made me drink ‘yellow rice wine’ to ‘do the month,’ but I don’t usually drink wine. I was told by an online doctor that a breastfeeding mother should not drink wine because alcohol can be passed on through my breast milk.* (P17, first-time mother)

Some women were told to supplement feeding with infant formula because they did not produce enough breast milk. This discouraged them from breastfeeding exclusively.

*In the first few days after delivery, my sister-in-law told me that I did not have sufficient breast milk and should supplement feeding with formula. But I thought all I needed to do was to keep the baby sucking, and the stimulation would help breast milk to come. She blamed me for starving my baby.* (P4, first-time mother)

#### Lack of facilities for breastfeeding in public

A lack of facilities for breastfeeding in public places caused mothers a great deal of inconvenience, since they felt embarrassed to breastfeed their baby in public.

*There are few breastfeeding rooms in the city. Not all public washrooms have a special place for changing diapers. I don’t think it is appropriate for me to breastfeed my baby in the washroom.* (P12, first-time mother)*.*

### Recommendations for breastfeeding promotion

Women openly expressed their need for support from professionals regarding breastfeeding. Lacking direct support from health professionals, they tapped into their social networks and the internet for information.

#### More breastfeeding information from professionals

The women stated that they had not received much support regarding breastfeeding from professionals. They sought support or answers from friends or the internet but were sometimes confused about conflicting suggestions.

*I was confused about whether I should use heat or cold therapy when I had mastitis. Somebody told me to use heat therapy, while others advised the use of cold therapy. I hope a professional can tell me which is right*. (P5, first-time mother)

#### Professional support at home

Women showed respect for and expectations of professional support. They hoped that postpartum home visit nurses could provide them with guidance on breastfeeding during their visits. They suggested that a breastfeeding consultant could make home visits to provide them with help.

*I hope that home visit nurses can promote lactation and support me through the difficulties of breastfeeding. It would be great if a breastfeeding consultant could make home visits.* (P3, first-time mother)

This theme clearly showed that women were in need of information and support from home visit nurses or a breastfeeding consultant.

## Discussion

This study explored the breastfeeding experiences of women during their early postpartum period in Shenzhen, China. Chinese women consider it natural to breastfeed their baby and regard it as a mission to do so [35, 36]. The women in this study were motivated to breastfeed their baby in a breastfeeding culture, but the difficulties they encountered negatively influenced exclusive breastfeeding during the early postpartum period.

The women expressed an intrinsic motivation to breastfeed and described it as a joyful experience that strengthened the emotional bond and degree of attachment between the mother and the infant. Chinese mothers are determined to “give the best” to their children and prioritize breastfeeding despite the difficulties, unconditionally sacrificing their own needs for the benefit of their children [37, 38]. Breastfeeding is seen as a moral obligation for a mother [20]. The findings of this study revealed that women made great efforts to promote or sustain the production of their breast milk, seeing it as one of the roles of a “good mother.” Mothers who had successful breastfeeding experiences during the early postpartum period were more likely to enjoy breastfeeding and to continue to breastfeed due to their enhanced breastfeeding self-efficacy [39, 40].

However, women also experienced many obstacles during their early breastfeeding experiences. In accordance with previous studies, first-time mothers in this study seemed to have more breastfeeding problems, such as difficulty latching, sore or cracked nipples, and mastitis [32]. However, there were two second-time mothers who were breastfeeding for the first time, and they seemed to report difficulties equal to those of the first-time mothers with regard to breastfeeding. Customized interventions should be provided for mothers according to their breastfeeding experience to support exclusive breastfeeding [41].

This study found that a lack of breastfeeding skills, a lack of public facilities, perceived insufficient breast milk, family members’ persuasion to supplement with infant formula, sore nipples, and pain are potential barriers to exclusive breastfeeding. These findings are consistent with previous research. These negative experiences of breastfeeding in the early postpartum period can impede the continuation of exclusive breastfeeding [42]. When faced with difficulties, women may have to choose infant formula instead of breastfeeding [23, 43]. According to this study, the most common reason for infant formula supplementation is perceived insufficient breast milk, which indicates low self-efficacy in relation to exclusive breastfeeding among Chinese women. Interestingly, this study also found that the attitude of relatives can influence Chinese women’s self-efficacy and behaviour with regard to exclusive breastfeeding [44]. Therefore, the women’s immediate social networks should be considered when designing breastfeeding promotion interventions for Chinese women [44].

In this study, women reported that family conflicts regarding the diet needed to promote breastfeeding were among the factors that influenced their breastfeeding experiences. Chinese people believe that the quality and quantity of breast milk affect the health of babies in the long run [36]. Hence, the women in this study paid a great deal of attention to their diet and consumed soups that were believed to promote lactation. Although women emphasized the benefits of breastfeeding for their babies, they also expressed concerns about their body weight. Women attempted to balance the costs to themselves with the benefits to their infant and subordinated the role of the self to that of “being a good mother” [45].

In a Chinese family, a woman is expected to submit to the opinions of her parents-in-law [46]. The grandparents of the newborn are responsible for providing the care required by postpartum mothers and newborns [47]. However, there may be intergenerational disagreements regarding what foods are good for young mothers and for lactation. New mothers usually have beliefs about nutritional supplements that differ from those of their mother or mother-in-law. The mothers prefer to obtain their information from social media and to receive support from their peers and professionals [48]. Therefore, breastfeeding diets and physical recovery may also need to be included in future breastfeeding education in China.

This study revealed a relationship between breastfeeding and postpartum depression. In this study, women reported that negative breastfeeding experiences, such as excessive pain due to cracked nipples, caused them to develop symptoms of depression. A previous study also reported reciprocal effects between breastfeeding and postpartum depression [49]. Negative effects on the mothers’ psychological wellbeing also resulted from low self-efficacy in breastfeeding, persuasion from family members to supplement feeding with infant formula, or the scarcity of social support for breastfeeding [50, 51].

Women explicitly expressed their need for timely information and support from health professionals with regard to breastfeeding. Without such support, they often found themselves searching the internet for information or seeking support from friends, but they sometimes received contradictory information that confused them. Recent randomized controlled trials found that m-health-based interventions were effective at promoting the exclusive breastfeeding rate at 6 months and the breastfeeding duration [48]. This might be considered a potential breastfeeding education format in the future.

It was notable that fewer than half of the interviewed mothers persisted in exclusive breastfeeding at 6 weeks postpartum. The exclusive breastfeeding rate among these women would be much lower at 6 months postpartum compared to the rate recommended by the WHO [4]. This finding might imply that breastfeeding promotion strategies need to be re-examined in this context. That is, in addition to a breastfeeding culture, more instrumental and emotional breastfeeding support should be provided to breastfeeding women. Women’s positive or negative breastfeeding experiences in the early postpartum period were also strong predictors of their continuation of breastfeeding [42].

In Shenzhen, new mothers in the early postpartum period receive visits from home visiting nurses. However, because most home visit nurses are not specialists in breastfeeding, they were unable to provide the women in our study with the guidance and support for breastfeeding that they needed and failed to solve the breastfeeding problems that the women encountered. A study in China also revealed that breastfeeding support services provided by health professionals fell far below the expectations of the mothers [23].

### Limitations

There are several limitations in this study. First, women were recruited in only one hospital, and the results of this study might be limited to the context of this study. Furthermore, the study focused only on women’s breastfeeding experiences in the first 6 weeks postpartum; these experiences might vary in different stages of breastfeeding. Second, the researcher who collected the data worked as a clinical nurse in the study hospital, although in a different department, which may have introduced some bias in this study. In the future, the effects of women’s early breastfeeding experiences on their decision regarding exclusive breastfeeding could be confirmed with a larger sample size and a more diverse group of participants.

## Conclusion

This study shows that breastfeeding is a dynamic process for new mothers. It provides an in-depth understanding of women’s breastfeeding experiences in the early postpartum period in a Chinese context. Breastfeeding mothers experience both joys and pains during their breastfeeding journey. However, insufficient breastfeeding knowledge, intergenerational disagreements regarding food supplements that may impede lactation, and a lack of professional support lead to problems beyond those of breastfeeding. Current breastfeeding services should be tailored to meet the needs of individual women. Home visit nurses, who make visits to postpartum women, should be provided with the necessary training to give advice and support for breastfeeding so that they can better serve breastfeeding women. Breastfeeding information and support should be given to both new mothers and significant members of their families to create a supportive environment in the home. With recent advances in internet platforms, it has been suggested that professional health advice and support be offered to postpartum women at home using an internet health platform operated by the relevant hospital or community health centre [48].

## Data Availability

The datasets used in the current study are available from the corresponding author upon reasonable request.

## Abbreviations

WHO: World Health Organization

## References

[1] Guo S, Fu X, Scherpbier RW, Wang Y, Zhou H, Wang X (2013). Breastfeeding rates in central and western China in 2010: implications for child and population health. Bull World Health Organ.

[2] Yang Z, Lai J, Yu D, Duan Y, Pang X, Jiang S (2016). Breastfeeding rates in China: a cross sectional survey and estimate of benefits of improvement. Lancet.

[3] World Health Organization (2013). Essential nutrition actions: improving maternal, newborn, infant and young child health and nutrition.

[4] World Health Organization (2014). Global targets indicators: what is measured gets done.

[5] Rollins NC, Bhandari N, Hajeebhoy N, Horton S, Lutter CK, Martines J (2016). Why invest, and what it will take to improve breastfeeding practices. Lancet.

[6] Liu P, Qiao L, Xu F, Zhang M, Wang Y, Binns CW (2013). Factors associated with breastfeeding duration: a 30-month cohort study in Northwest China. J Hum Lact.

[7] Xu F, Qiu L, Binns CW, Liu X (2009). Breastfeeding in China: a review. Int Breastfeed J.

[8] Pang WW, Aris IM, Fok D, Soh SE, Chua MC, Lim SB (2016). Determinants of breastfeeding practices and success in a multi-ethnic Asian population. Birth.

[9] Qiu L, Zhao Y, Binns CW, Lee AH, Xie X (2009). Initiation of breastfeeding and prevalence of exclusive breastfeeding at hospital discharge in urban, suburban and rural areas of Zhejiang China. Int Breastfeed J.

[10] Lai JQ, Yin SA, Ma GS, Piao JH, Yang XG. The survey of newborn status and feeding of infants and young children in china. Acta Nutrimenta Sinica. 2006;28:5.

[11] Tang L. A cohort study of infant feeding practices and maternal breastfeeding problems in a rural area of Sichuan Province, PR China. Curtin University; 2013.

[12] Shrimpton R. Early initiation of breastfeeding: World Health Organization; 2017. Available from: https://www.who.int/elena/titles/commentary/early_breastfeeding/en/. Accessed 9 Apr 2019

[13] Takahashi K, Ganchimeg T, Ota E, Vogel JP, Souza JP, Laopaiboon M (2017). Prevalence of early initiation of breastfeeding and determinants of delayed initiation of breastfeeding: secondary analysis of the WHO global survey. Sci Rep.

[14] Zhou Q, Younger KM, Kearney JM (2010). An exploration of the knowledge and attitudes towards breastfeeding among a sample of Chinese mothers in Ireland. BMC Public Health.

[15] McAndrew F, Thompson J, Fellows L, Large A, Speed M, Renfrew MJ (2012). Infant feeding survey 2010.

[16] Feenstra MM, Jorgine KM, Thygesen M, Danbjorg DB, Kronborg H (2018). Early breastfeeding problems: a mixed method study of mothers' experiences. Sex Reprod Health.

[17] Odom EC, Li R, Scanlon KS, Perrine CG, Grummer SL (2013). Reasons for earlier than desired cessation of breastfeeding. Pediatrics.

[18] Wagner EA, Chantry CJ, Dewey LA (2013). Nommsen-Rivers breastfeeding concerns at 3 and 7 days postpartum and feeding status at 2 months. Pediatrics.

[19] Spencer RL, Greatrex-White S, Fraser DM (2015). ‘I thought it would keep them all quiet’. Women's experiences of breastfeeding as illusions of compliance: an interpretive phenomenological study. J Adv Nurs.

[20] Fox R, McMullen S, Newburn M (2015). UK women’s experiences of breastfeeding and additional breastfeeding support: a qualitative study of baby Café services. BMC Pregnancy Childbirth.

[21] Demirtas B, Ergocmen B, Taskin L (2012). Breastfeeding experiences of Turkish women. J Clin Nurs.

[22] Borra C, Iacovou M, Sevilla A (2015). New evidence on breastfeeding and postpartum depression: the importance of understanding women’s intentions. Matern Child Health J.

[23] Zhang K, Tang L, Wang H, Qiu L-Q, Binns C, Lee A (2015). Why do mothers of young infants choose to formula feed in China? Perceptions of mothers and hospital staff. Int J Environ Res Public Health.

[24] Tang L, Zhu R, Zhang X (2016). Postpartum depression and social support in China: a cultural perspective. J Health Commun.

[25] Robinson H, Buccini G, Curry L, Perez-Escamilla R. The World Health Organization Code and exclusive breastfeeding in China, India, and Vietnam. Matern Child Nutr. 2019;15(1):e12685. 10.1111/mcn.12685 PMC7199093 30194804

[26] Ku CM, Chow SK (2010). Factors influencing the practice of exclusive breastfeeding among Hong Kong Chinese women: a questionnaire survey. J Clin Nurs.

[27] Loke AY, Chan LKS (2013). Maternal breastfeeding self-efficacy and the breastfeeding behaviors of newborns in the practice of exclusive breastfeeding. J Obstet Gynecol Neonatal Nurs.

[28] Baxter P, Jack S (2008). Qualitative case study methodology: study design and implementation for novice researchers. Qual Rep.

[29] Shenzhen Statistic Bureau and NBS Survey Office in Shenzhen (2017). Shenzhen statistical yearbook.

[30] Galipeau R, Baillot A, Trottier A, Lemire L (2018). Effectiveness of interventions on breastfeeding self-efficacy and perceived insufficient milk supply: a systematic review and meta-analysis. Matern Child Nutr.

[31] Sheehan A, Schmied V, Barclay L (2010). Complex decisions: theorizing women’s infant feeding decisions in the first 6 weeks after birth. J Adv Nurs.

[32] Yang X, Ip WY, Gao LL (2018). Maternal intention to exclusively breastfeed among mainland Chinese mothers: a cross-sectional study. Midwifery.

[33] Mayring P (2000). Qualitative content analysis. Forum Qual Soc Res.

[34] Creswell J (2014). Research design: qualitative, quantitative and mixed methods approaches.

[35] Zhang Y, Jin Y, Vereijken C, Stahl B, Jiang H (2018). Breastfeeding experience, challenges and service demands among Chinese mothers: a qualitative study in two cities. Appetite.

[36] Chen WL (2010). Understanding the cultural context of Chinese mothers’ perceptions of breastfeeding and infant health in Canada. J Clin Nurs.

[37] Leung JT, Shek DT (2011). “All I can do for my child”–development of the Chinese parental sacrifice for Child’s education scale. Int J Disabil Hum Dev.

[38] Leung JT, Shek DT (2013). Parental beliefs and parental sacrifice of Chinese parents experiencing economic disadvantage in Hong Kong: implications for social work. Br J Soc Work.

[39] Henshaw EJ, Fried R, Siskind E, Newhouse L, Cooper M (2015). Breastfeeding self-efficacy, mood, and breastfeeding outcomes among primiparous women. J Hum Lact.

[40] Schafer EJ, Campo S, Colaizy TT, Mulder PJ, Breheny P, Ashida S (2017). First-time mothers’ breast-feeding maintenance: role of experiences and changes in maternal perceptions. Public Health Nutr.

[41] Huang Y, Ouyang YQ, Redding SR (2019). Previous breastfeeding experience and its influence on breastfeeding outcomes in subsequent births: a systematic review. Women Birth.

[42] DiGirolamo A, Thompson N, Martorell R, Fein S, Grummer-Strawn L (2005). Intention or experience? Predictors of continued breastfeeding. Health Educ Behav.

[43] Ouyang YQ, Su M, Redding SR (2016). A survey on difficulties and desires of breast-feeding women in Wuhan, China. Midwifery.

[44] Bai DL, Fong DYT, Lok KYW, Tarrant M (2015). Relationship between the infant feeding preferences of Chinese mothers' immediate social network and early breastfeeding cessation. J Hum Lact.

[45] Tully KP, Ball HL (2013). Trade-offs underlying maternal breastfeeding decisions: a conceptual model. Matern Child Nutr.

[46] Siu BW, Leung SS, Ip P, Hung SF, O'Hara MW (2012). Antenatal risk factors for postnatal depression: a prospective study of Chinese women at maternal and child health centres. BMC Psychiatry.

[47] Chang SHC, Hall WA, Campbell S, Lee L (2018). Experiences of Chinese immigrant women following “Zuo Yue Zi” in British Columbia. J Clin Nurs.

[48] Alianmoghaddam N, Phibbs S, Benn C (2019). “I did a lot of Googling”: a qualitative study of exclusive breastfeeding support through social media. Women Birth.

[49] Pope CJ, Mazmanian D (2016). Breastfeeding and postpartum depression: an overview and methodological recommendations for future research. Depress Res Treat.

[50] Yang X, Gao LL, Ip WY, Chan WCS (2016). Predictors of breast feeding self-efficacy in the immediate postpartum period: a cross-sectional study. Midwifery.

[51] Gökçeoğlu E, Küçükoğlu S (2017). The relationship between insufficient milk perception and breastfeeding self-efficacy among Turkish mothers. Glob Health Promot.

